# Wolves in Bhutan Are Part of the Ancient Himalayan Wolf (*Canis lupus chanco*) Lineage

**DOI:** 10.1002/ece3.74201

**Published:** 2026-08-19

**Authors:** Tashi Dhendup, Antonio Sampedro, Joshua J. Robinson, Jan E. Janečka, Geraldine Werhahn

**Affiliations:** ^1^ School of the Environment The University of Queensland Queensland Australia; ^2^ Department of Forests and Park Services Ministry of Energy and Natural Resources, Royal Government of Bhutan Thimphu Bhutan; ^3^ Himalayan Wolves Project, Himalayan Carnivore Foundation St. Gallen Switzerland; ^4^ Biodiversity Research Institute (CSIC—University of Oviedo—Principality of Asturias) Mieres Spain; ^5^ Department of Biological Sciences Duquesne University Pittsburgh Pennsylvania USA; ^6^ Wildlife Conservation Research Unit (WildCRU), Department of Biology University of Oxford Oxford UK; ^7^ IUCN SSC Canid Specialist Group Oxford UK

**Keywords:** Asia, Bhutan, biodiversity, Himalayan wolf, wildlife

## Abstract

Three wolf lineages are recognised in South Asia, which include the Himalayan wolf ( *Canis lupus chanco* ), inhabiting the high Himalayas and the Tibetan Plateau; the Indian wolf ( *Canis lupus pallipes* ), found on the Indian subcontinent; and the Holarctic grey wolf, represented by multiple *Canis lupus* subspecies, found in adjacent regions of Central Asia. The Himalayan wolf represents an evolutionarily distinct high‐elevation wolf lineage, yet genetic information remains scarce in the eastern Himalaya, including Bhutan, constituting a critical knowledge gap. During Bhutan's nationwide snow leopard survey (2022–2023), putative snow leopard scats were collected and a subset was genetically confirmed as *C. lupus* . We extracted DNA and amplified the mitochondrial *control region* (D‐loop) and cytochrome *b* sequences from 13 samples (Jigme Dorji National Park: *n* = 1; Wangchuck Centennial National Park: *n* = 12). Phylogenetic analyses placed all Bhutanese sequences within the monophyletic Himalayan wolf clade, and median‐joining haplotype networks provided concordant support for this placement. All detected haplotypes matched those previously reported from northwestern Nepal, indicating that Bhutanese wolves fall within the known Himalayan wolf mtDNA clade. These results provide the first genetic evidence that the Himalayan wolf lineage is present in Bhutan and provide an initial assessment of mtDNA diversity. Expanded non‐invasive sampling across Bhutan, including areas with wolf records but lacking genetic representation (e.g., Jigme Khesar Strict Nature Reserve and Paro Forest Division), coupled with individual identification and nuclear markers, will be necessary to quantify genetic diversity and population connectivity, and provide first population size estimates. Such sampling will be critical for assessing fine‐scale population structure and informing national conservation planning for this threatened wolf lineage endemic to High Asia.

## Graphical abstract

During Bhutan's nationwide snow leopard survey (2022–2023), putative snow leopard scat samples were collected, of which some were genetically identified as belonging to the wolf. Mitochondrial DNA analyses placed all Bhutanese sequences within the monophyletic Himalayan wolf clade and matched haplotypes previously reported from Nepal. These findings provide the first genetic confirmation of the Himalayan wolf in Bhutan and an initial assessment of its mitochondrial diversity.

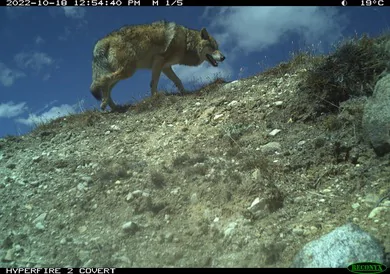

## 1 Introduction

The Himalayan wolf ( *Canis lupus chanco* ) is an apex predator of the high Himalayas and the Tibetan Plateau and is listed as Vulnerable on the IUCN Red List across its global range due to its low population size and declining numbers (Werhahn et al. 2024). The Himalayan wolf is widely regarded as one of the most evolutionarily distinct and ancient wolf lineages in Asia. Genetic evidence, particularly mitochondrial DNA (mtDNA), complemented by nuclear markers, indicates that this lineage forms a monophyletic clade basal to the widespread wolf‐dog (Holarctic grey wolf/domestic dog) complex, consistent with deep, ancient divergence (Aggarwal et al. 2007; Hennelly et al. 2025; Sharma et al. 2004; Werhahn et al. 2017). Estimates of divergence time vary across datasets and calibrations, but mtDNA‐based analyses commonly place the split hundreds of thousands of years ago, with some studies suggesting an older separation of 0.35–0.74 million years (Hennelly 2022; Sharma et al. 2004; Werhahn et al. 2018). Beyond mtDNA, evidence from sex‐chromosome markers (e.g., ZF loci) and nuclear single‐nucleotide polymorphisms (SNPs) associated with the hypoxia pathway supports functional specialisation for high‐elevation, low‐oxygen environments (Liu et al. 2019; Signore et al. 2019; Werhahn et al. 2018; Zhang et al. 2014). Additional phenotypic differentiation (including reported howl‐acoustic divergence and cranial variation) has also been noted (Hennelly et al. 2017; Shrotriya et al. 2012; Viranta et al. 2025; Werhahn et al. 2020, 2018, 2022).

**FIGURE 1:**
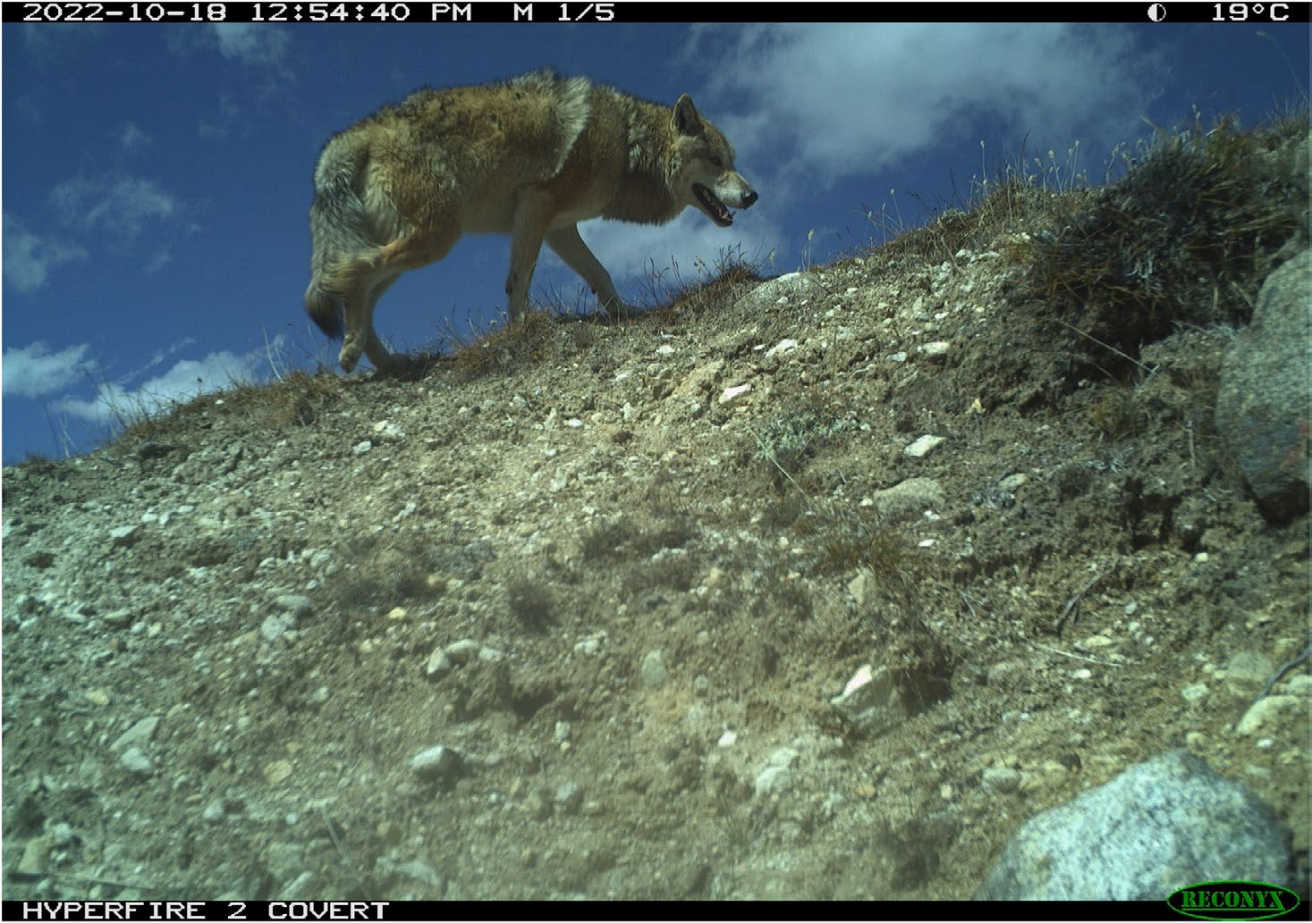
A Himalayan wolf ( *Canis lupus chanco* ) camera‐trapped in central Bhutan during the Bhutan nationwide snow leopard survey in 2022–2023 (elevation: 4313 m). *Source*: DoFPS, Bhutan.

Bhutan is located along the south‐eastern edge of the Himalayan wolf distribution range, which comprises the Himalayas and the Tibetan Plateau. It is suspected that the Himalayan wolf lineage is also found in Bhutan (Figure 1). To the west of Bhutan, wolves in the Indian state of Sikkim, known from limited localities, have been genetically confirmed as Himalayan wolves, whereas in Arunachal Pradesh, along the India–China border, they have not been genetically assessed (Choudhury 2015; Joshi et al. 2020). To the north, connectivity is established via high‐altitude passes, facilitating wildlife movement, as confirmed by gene flow among snow leopards ( *Panthera uncia* ) in Bhutan, Tibet and Nepal (Dhendup et al. 2025). Consequently, wolves occurring in Bhutan's high‐altitude landscapes may be connected via dispersal to other regional populations across these borders and could belong to the Himalayan wolf lineage; however, the subspecific identity of Bhutan's wolves remains unresolved in the absence of targeted genetic sampling and systematic phylogeographic assessment within Bhutan.

**FIGURE 2:**
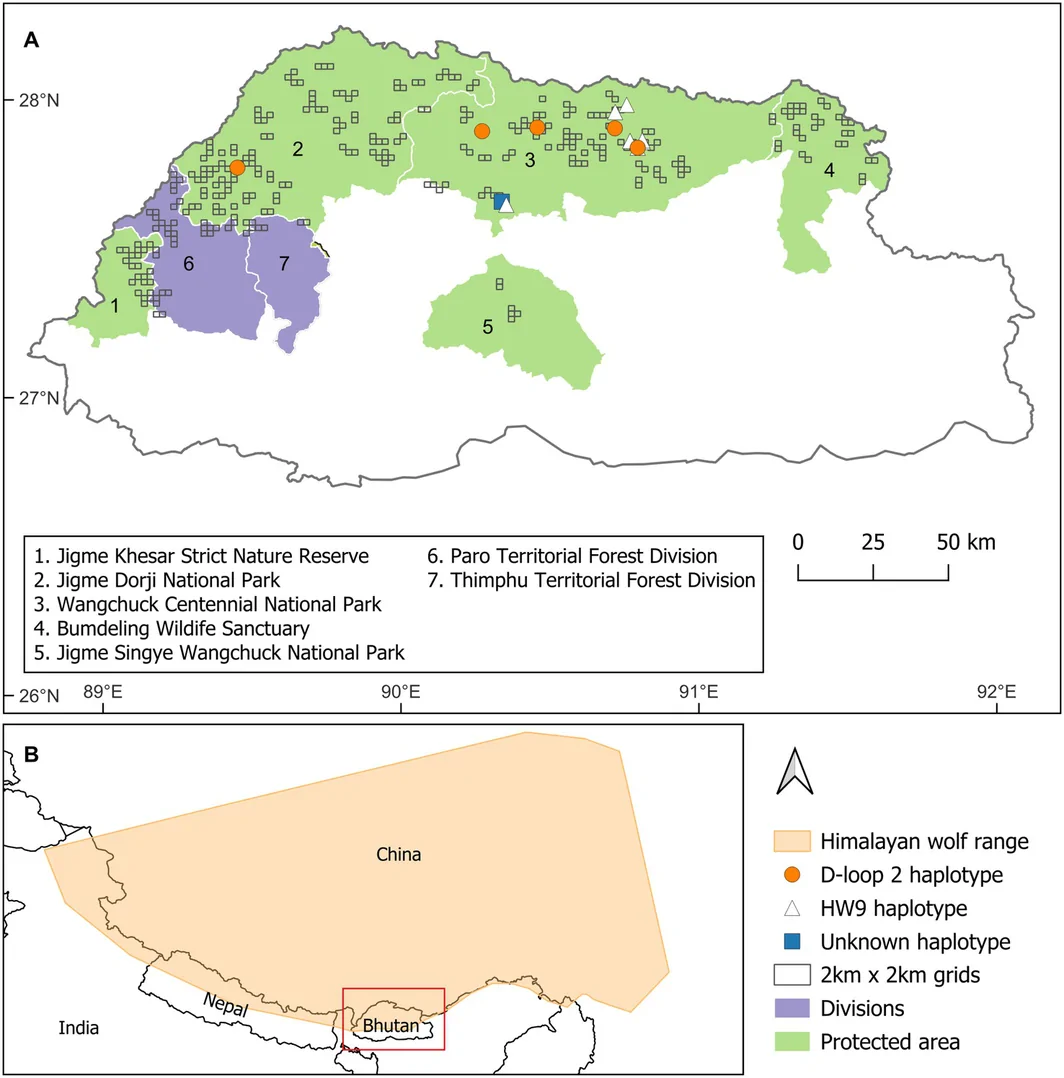
(A) Map of the nationwide snow leopard survey area in Bhutan during 2022–2023, showing the 2 × 2 km sampling grid and locations of wolf‐positive samples with observed D‐loop haplotypes (*D‐loop2*, *HW9*, or unknown). (B) Location of Bhutan within the geographic range of the Himalayan wolf, based on distribution data from the IUCN Red List (Werhahn et al. 2024).

Wolves remain one of Bhutan's least‐studied large carnivores and are known from only a limited number of areas, including Wangchuck Centennial National Park (WCNP), Jigme Dorji National Park (JDNP), Jigme Khesar Strict Nature Reserve (JKSNR) and Paro Forest Division (PFD) (Dhendup et al. 2024; Figure 2). Anecdotal accounts suggest the species may have been close to extirpation in the past; however, recent observations indicate a gradual resurgence. This is reflected in increasingly frequent reports of human–wildlife conflict in high‐altitude landscapes, where wolves face threats resulting from livestock depredation alongside snow leopards (BBS 2024; Jamtsho and Katel 2019). Despite these concerns, dedicated field studies and systematic monitoring are largely absent. Opportunistic ‘bycatch’ detections from camera‐trap surveys targeting other species suggest that wolves may currently occupy less than 7% of the country (Dhendup et al. 2024). To ensure its continued recovery, the species has been afforded protection under Schedule II of the Forest and Nature Conservation Act of Bhutan 2023 (RGoB 2023). However, Bhutan currently lacks a dedicated national programme focused on wolf research, conflict mitigation, or conservation planning.

We obtained scat samples during Bhutan's second national snow leopard survey that were genetically identified as *Canis lupus* (NCD 2023). Using DNA extracted from faecal samples, we sequenced the mitochondrial *control region* (D‐loop) and cytochrome *b* and, for the first time, described extant mitochondrial lineages of wolves in Bhutan.

## 2 Materials and Methods

### 2.1 Study Area and Sample Collection

Bhutan conducted a systematic, nationwide snow leopard survey in 2022–2023, covering all potential snow leopard habitats across the country. A 2 km × 2 km grid was overlaid across elevations of 3400–5200 m asl, and after accounting for accessibility, 310 grid cells were selected for camera‐trap deployment across JKSNR, PFD, JDNP, WCNP, Bumdeling Wildlife Sanctuary, Thimphu Forest Division and Jigme Singye Wangchuck National Park (NCD 2023; Figure 2).

In parallel with camera trapping, putative snow leopard scats were collected during camera trap installation, monitoring and retrieval along travel routes and at camera trap sites to assess genetic diversity and population structure (Dhendup et al. 2025). In total, 184 putative snow leopard scats were collected and preserved in plastic vials containing silica desiccant. To minimise contamination, field protocols required wearing a fresh pair of gloves for each scat sample. Samples were then transported to a dedicated genetics laboratory at Duquesne University in the U.S., where DNA extraction and subsequent laboratory analyses were conducted.

### 2.2 Laboratory Analysis of Genetic Wolf Samples

Initial DNA metabarcoding of the scat samples (*n* = 184) was conducted to identify the origin of each scat and potential prey consumed. An approximately 100‐bp fragment of the mitochondrial *12S ribosomal RNA* (MT‐*RNR1*) was chosen as the DNA barcode because of its widespread use for taxonomic identification of mammal and bird scat (Riaz et al. 2011; van der Heyde et al. 2021). PCR reactions contained 1 μL DNA template (extracted using the Qiagen QIAamp Fast DNA Stool Mini kit), 5 μL KAPA HIFI HotStart Ready Mix (2×), 0.5 μL of the forward and reverse *12SV5* primer (20 μM; Riaz et al. 2011) and 3 μL PCR‐grade water. Each primer had the Illumina adapter on the 5′ end to enable indexing as described below. PCRs were performed under the following conditions: denaturation at 95°C for 3 min, followed by 40 cycles of 95°C for 30 s, 60°C for 30 s and 72°C for 30 s, with a final extension at 72°C for 5 min. Products were confirmed via gel electrophoresis. Amplicons were purified with Agencourt AMPure XP beads (Beckman Coulter Inc., Brea, CA, USA). An indexing PCR was used to incorporate unique barcodes to each sample via a Nextera XT index kit (Illumina Inc., San Diego, CA, USA). PCR reactions contained 5 μL DNA template, 12.5 μL KAPA HIFI HotStart Ready Mix (2×), 2.5 μL of forward index primer, 2.5 μL of reverse index primer and 2.5 μL PCR‐grade water. PCRs were performed under the following conditions: denaturation at 95°C for 3 min, followed by 10 cycles of 95°C for 30 s, 55°C for 30 s and 72°C for 30 s, with a final extension at 72°C for 5 min. Products were purified with Agencourt AMPure XP beads, run on an agarose gel and stained with GelGreen. Samples were pooled and diluted to a concentration of 4 nM. The library was loaded and sequenced on an Illumina MiSeq using a paired‐end run with the MiSeq 2 x 250 bp Reagent Micro Kit V2 at the Janecka Genomics Laboratory.

After sequencing, the FASTQ sequences were demultiplexed, adapters were removed and the reads were imported into CLC Genomic Workbench v22 (Qiagen Bioinformatics, Redwood City, CA, USA) for preliminary analysis. Raw reads were quality‐checked and trimmed with a quality score cutoff of 0.05. De novo assembly was conducted using the following parameters: a mismatch cost of 2, an insertion cost of 2, a deletion cost of 3, a minimum contig length of 90 bp, a 90% length fraction and a 94% similarity threshold. Nonspecific matches were mapped randomly. Consensus sequences for each contig with at least 500 reads were extracted and identified using NCBI's Basic Local Alignment Search Tool (BLAST; https://blast.ncbi.nlm.nih.gov/Blast.cgi). Contigs were assigned to the species with the highest max identity in the GenBank database, provided the max identity was at least 97%. In cases with multiple carnivore species identified, the carnivore with the largest read count was assigned to be the source species. However, no scats contained both snow leopard and *Canis* spp. reads. The species of origin for 24 scats was identified as *Canis* spp. in the initial analysis because the MT‐*RNR1* barcode could not distinguish between domestic dogs ( *Canis lupus familiaris* ) and Himalayan wolves ( *Canis lupus chanco* ).

The 24 scats produced by *Canis* spp. were reanalysed in an additional metabarcoding run. In the additional run, the MT‐*RNR1* barcode and two other loci were amplified. The MT‐*RNR1* barcode was amplified under the same conditions as the first run. The two other loci included a 508‐bp segment of the cytochrome *b* and a 242‐bp segment of the *control region* (D‐loop) using primers previously used in a phylogenetic analysis of Himalayan wolves (Ghazali et al. 2016; Werhahn et al. 2017). All three markers were amplified in separate PCRs. PCR reactions for the cytochrome *b* and D‐loop loci contained 2 μL DNA template, 14 μL KAPA HIFI HotStart Ready Mix (2×), 2 μL of each primer (10 μM) with the Illumina indexing overhang in the 5′ end, and 0.1 μL bovine serum albumin (10 mg/mL). The cytochrome *b* PCR was performed under the following conditions: denaturation at 95°C for 5 min, followed by 35 cycles of 95°C for 30 s, 50°C for 90 s and 72°C for 30 s, with a final extension at 72°C for 10 min. The D‐loop PCR was performed under the following conditions: denaturation at 95°C for 5 min, followed by 40 cycles of 95°C for 30 s, 67°C for 30 s and 72°C for 30 s, and a final extension at 72°C for 10 min. Products were visualised, purified, indexed and pooled to 4 nM as previously described. The three pools were combined (95% MT‐*RNR1*, 2.5% cytochrome *b*, 2.5% D‐loop), indexed according to the protocol described above, and sequenced on the MiSeq.

FASTQ sequences were imported into CLC Genomic Workbench, and the MT‐*RNR1* reads were analysed as previously described to ensure consistency between runs. The cytochrome *b* and D‐loop reads were mapped to the Himalayan wolf reference sequences (GenBank accession numbers: KY996534 (cytochrome *b*) and NC_010340 (D‐loop)) using the previously described parameters, except that the length fraction was 40% and the similarity was 90%. The consensus sequences were extracted and run against the GenBank database to determine if a sample contained either Himalayan wolf or domestic dog reads. Identification was made if the consensus sequences contained at least 97% similarity with the top hit in the database. To acquire complete coverage for cytochrome *b*, we purified the PCR products of individuals identified as Himalayan wolves using ZR DNA Sequencing Clean‐up Kits (Zymo Research, Irvine, CA, USA) and sent them to be sequenced via Sanger sequencing on an Applied Biosystems 3730xl instrument at the Biotechnology Resource Center at Cornell University, NY, USA.

### 2.3 Phylogenetic Analysis of mtDNA

To identify unique D‐loop and cytochrome *b* haplotypes in Himalayan wolves from Bhutan, we utilised the *pegas* (version 1.3) and *ape* (version 5.8‐1) packages in R (Paradis 2010; Paradis and Schliep 2019). The D‐loop and cytochrome *b* sequences were imported as DNAbin objects, and haplotypes were identified using the *haplotype()* function, assigning each haplotype to the geographic origin of the sampled individuals.

To contextualise the phylogeographic position of our samples, we incorporated publicly available reference sequences from the NCBI GenBank database (Table S1), including 30 D‐loop and 11 cytochrome *b* sequences from canids in Central Asia, such as the Tibetan fox ( *Vulpes ferrilata* ) and golden jackal ( *C. aureus* ), as well as coyote ( *C. latrans* ) from North America, domestic dog and Holarctic grey wolves ( *C. lupus* sp.) worldwide. The reference dataset comprised a mixture of targeted mitochondrial fragments (D‐loop and cytochrome *b*) and complete mitogenomes. When required, sections that aligned with the sequences we generated were extracted from whole mitochondrial genomes using the *matchPattern()* function from the *Biostrings* package, version 2.78 (Pagès et al. 2025). For this step, we used published Himalayan wolf reference sequences as templates GenBank accession numbers: KY996530.1 (D‐loop) and KY996533.1 (cytochrome *b*).

Sequence alignment was performed using ClustalW and MUSCLE v3 (Chenna et al. 2003; Edgar 2004; Veen and Hoekstra 2020). We compared alignment quality metrics between the two methods and observed high agreement (99.6% similarity in pairwise distances). To ensure methodological consistency with previous analyses of Himalayan wolf mitochondrial haplotypes, we used the ClustalW alignments for downstream processing (Werhahn et al. 2017).

Aligned sequences were trimmed to a consensus length of 242‐bp for D‐loop and 508‐bp for cytochrome *b* (Werhahn et al. 2017). Haplotype networks were constructed using the Median‐Joining algorithm (Bandelt et al. 1999), as implemented in PopART version 1.7 (Leigh et al. 2015). Phylogenetic relationships were reconstructed separately for the 508‐bp cytochrome *b* and 242‐bp D‐loop fragments using maximum likelihood in IQ‐TREE v2. Model selection was conducted with ModelFinder under the Bayesian Information Criterion (BIC). The best‐fitting models were TPM2u + F + G4 for *cytochrome b* and K2P + G4 for D‐loop. Node support was evaluated using 1000 ultrafast bootstrap replicates and 1000 SH‐aLRT tests, and trees were midpoint rooted and visualised in FigTree v1.4.4.

### 2.4 Estimating Genetic Diversity

We delineated putative individuals by treating samples sharing the same haplotype as the same individual only when collected within 50 km of each other; identical haplotypes detected > 50 km apart were conservatively assigned to different individuals. We then quantified haplotype diversity from haplotype assignments using an unbiased estimator (Nei 1987), implemented via a custom function applied separately to cytochrome *b* and D‐loop. To evaluate the polymorphism of the selected regions, we calculated nucleotide diversity (π) directly from the sequence alignments used for canid sample comparison in the haplotype network. We used the function *nuc.div()* from the R package pegas (Paradis 2010), which computes the mean proportion of pairwise nucleotide differences among sequences. To further characterise sequence divergence, pairwise nucleotide differences were calculated with the function *dist. dna()* in the ape package (Paradis and Schliep 2019) using the raw distance model (‘N’) and pairwise deletion of missing sites, thus generating full distance matrices for both regions across the canid samples.

## 3 Results

### 3.1 Genetic Diversity

We successfully amplified mtDNA markers from 13 wolf scats (1 from JDNP and 12 from WCNP), yielding usable sequences for 12 samples at the D‐loop and 11 samples at cytochrome *b*. We identified two D‐loop haplotypes and one cytochrome *b* haplotype (Table S2). All 11 cytochrome *b* sequences were identical, indicating no within‐sample haplotypic variation (Hd = 0) and matched the haplotype previously reported by Werhahn et al. (2017) from Upper Humla in northwestern Nepal (GenBank accession number: KY996533). In contrast, D‐loop variation was higher: five samples matched the *Himalayan wolf D‐loop 2* haplotype (NCBI GenBank Accession KY996530), and seven matched *HW9_Museum Nepal* (NCBI GenBank Accession AY333738).

Applying our conservative haplotype–distance rule, we delineated five putative individuals from the 13 mtDNA‐positive samples. The D‐loop exhibited moderate haplotype diversity (Hd = 0.5303) and a mean nucleotide difference of 5.36% across aligned sites.

### 3.2 Phylogeography

**FIGURE 3:**
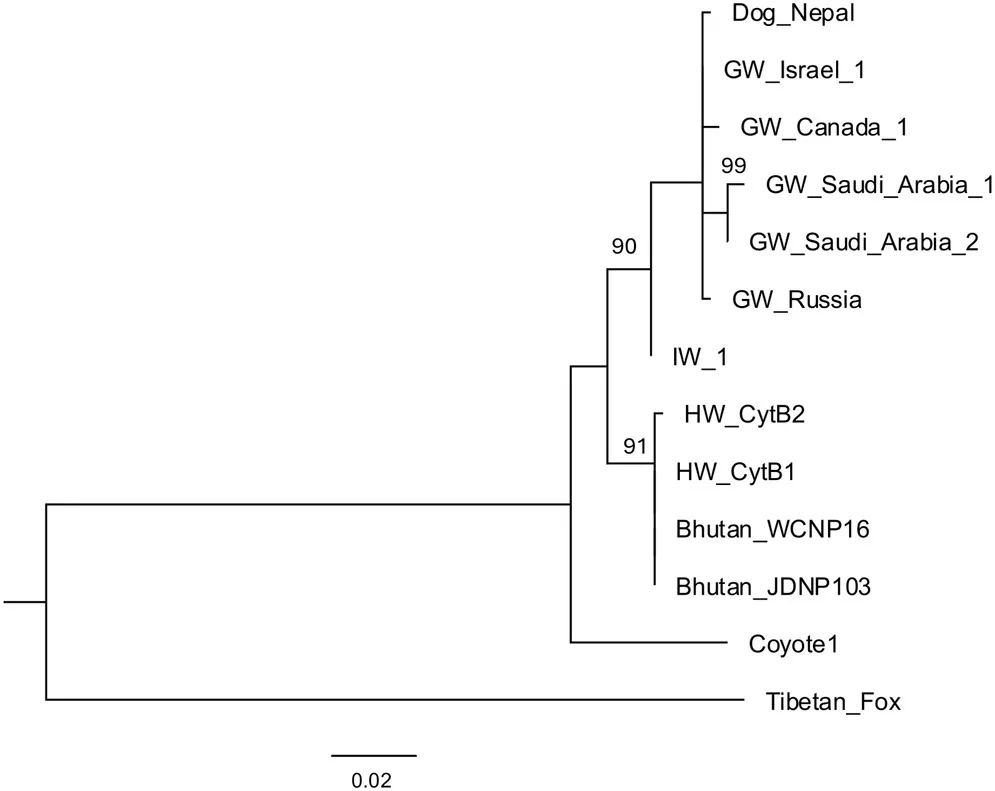
Maximum‐likelihood phylogeny of the 508‐bp cytochrome *b* fragment reconstructed in IQ‐TREE v2 (GW: Grey Wolf, HW: Himalayan Wolf, IW: Indian Wolf).

The mtDNA sequences matched known haplotypes within the maternal Himalayan wolf lineage. In the cytochrome *b* phylogeny, the Bhutan samples formed a strongly supported clade with previously described Himalayan wolf haplotypes and were nearly identical to sequences from Tibet and Nepal, clearly separating them from other wolf lineages (Figure 3).

**FIGURE 4:**
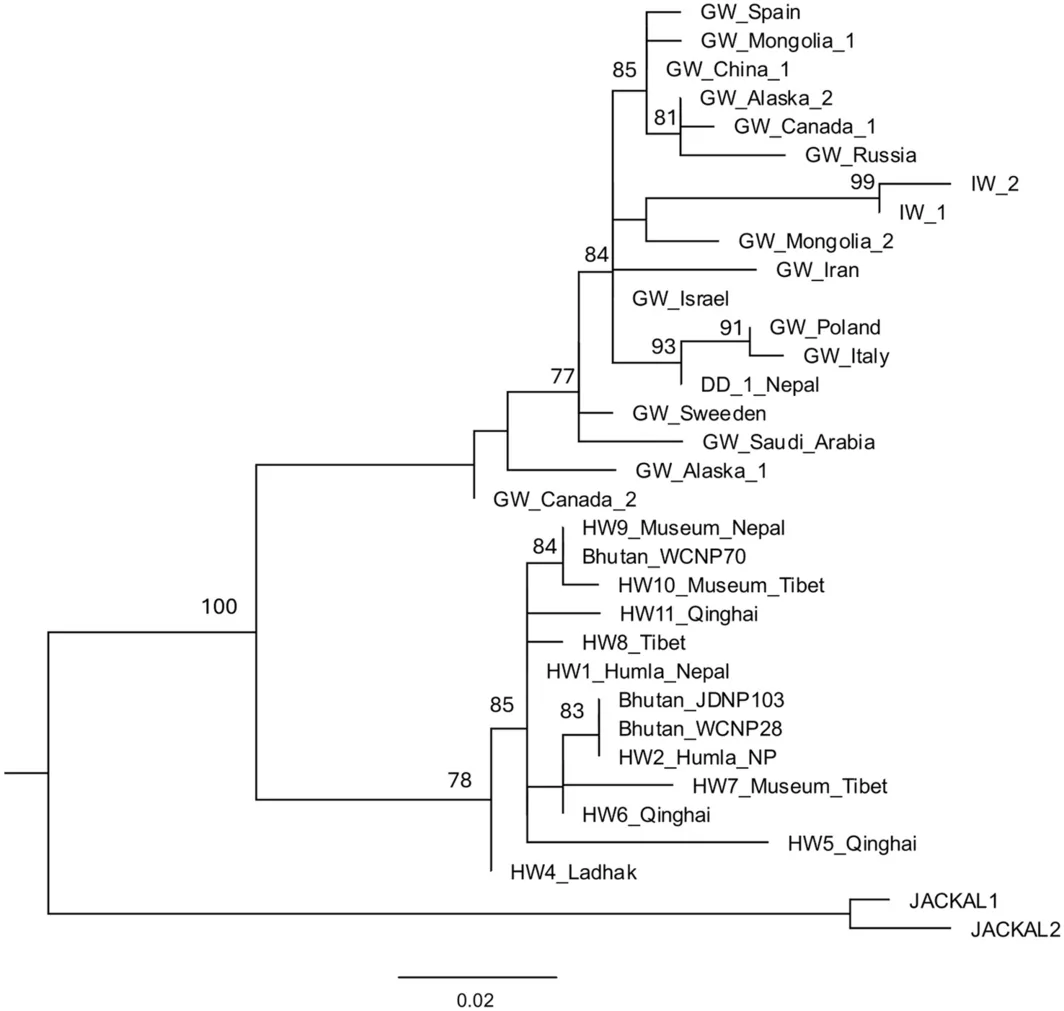
Maximum‐likelihood phylogeny of the 242‐bp *control region* (D‐loop) fragment reconstructed in IQ‐TREE v2. DD, domestic dog; GW, grey wolf; HW, Himalayan wolf; IW, Indian wolf.

Although the D‐loop showed greater sequence variation and lower support at some shallow nodes, its overall topology was consistent with that of the cytochrome *b* results. The D‐loop phylogeny likewise placed Bhutan sequences within the Himalayan wolf clade, clustering them with samples from Tibet, Nepal and Qinghai, and provided strong support for separating them from other wolf lineages (Figure 4). The *Himalayan wolf D‐loop 2* haplotype was first described by Werhahn et al. (2017) from northwestern Nepal (GenBank accession number: KY996530), while the *HW9_Museum Nepal* haplotype (GenBank accession number: AY333738) was first described by Sharma et al. (2004) from a museum specimen collected in Nepal.

**FIGURE 5:**
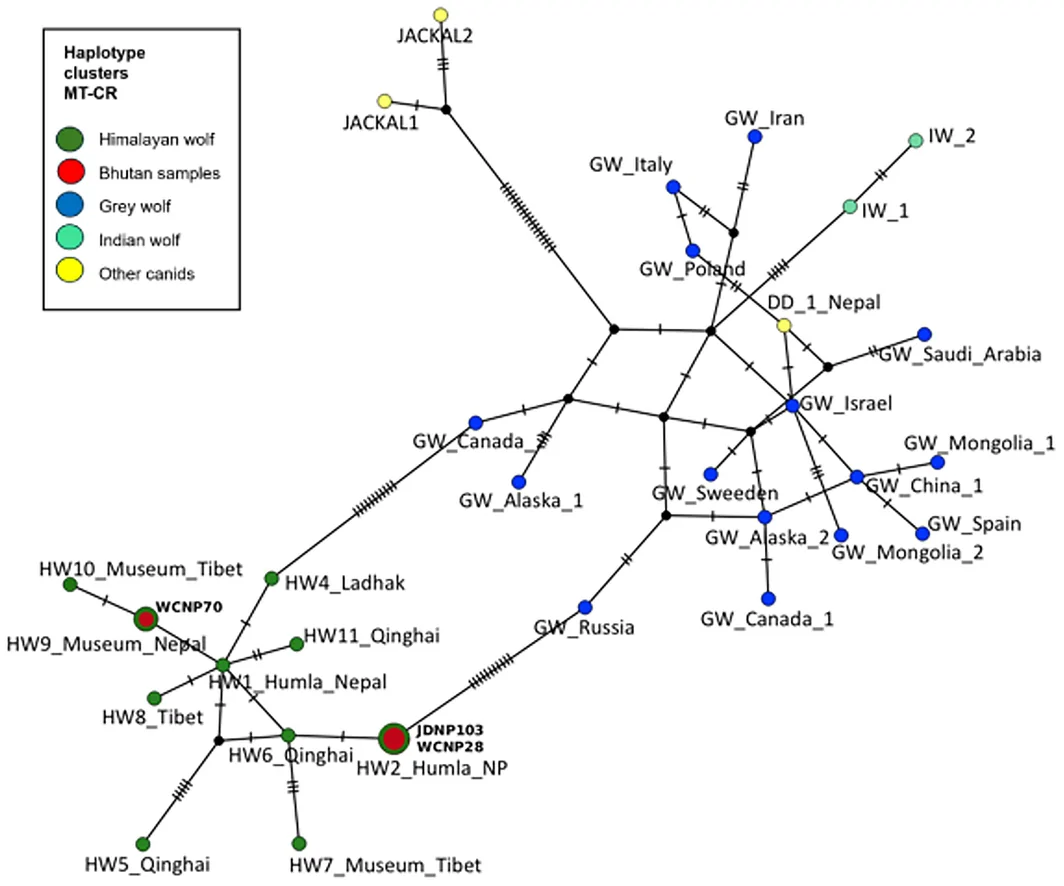
Median‐joining network based on 242 bp D‐loop haplotypes of canids. The relative position of wolves from Bhutan, based on current research samples, is highlighted in red. Each circle represents a different haplotype, and its size is proportional to the number of samples that share it. DD, domestic dog; GW, grey wolf; HW, Himalayan wolf; IW, Indian wolf.

**FIGURE 6:**
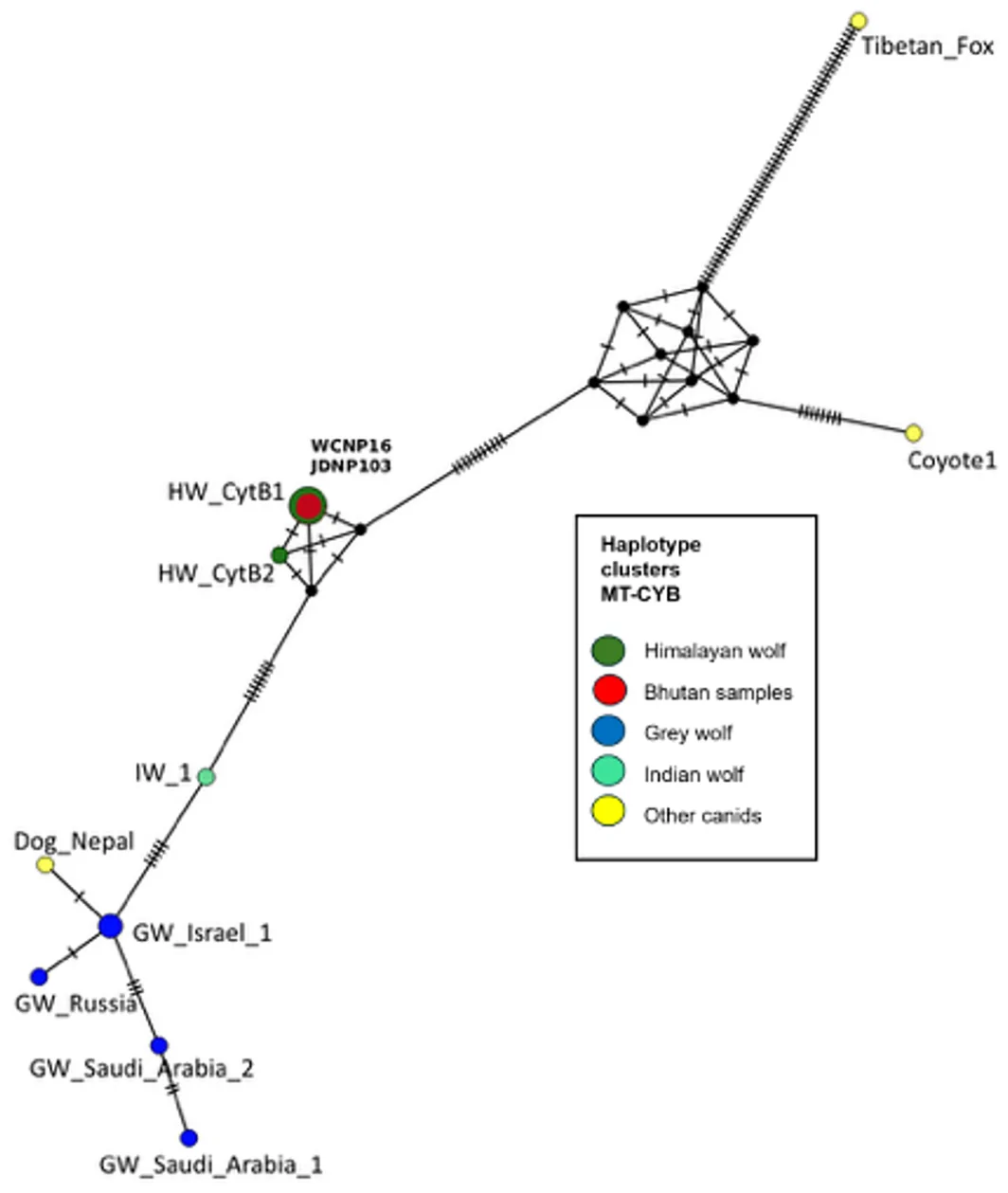
Median‐joining network based on 508 bp cytochrome *b* haplotypes of canids. The relative position of wolves from Bhutan, based on current research samples, is highlighted in red. Each circle represents a different haplotype, and its size is proportional to the number of samples that share it. DD, domestic dog; GW, grey wolf; HW, Himalayan wolf; IW, Indian wolf.

The median‐joining haplotype network corroborated these phylogenetic results, placing all scat‐derived sequences within the Himalayan wolf cluster. The D‐loop network further shows that Himalayan wolf haplotypes form a distinct cluster, and the two haplotypes detected in JDNP and WCNP represent different maternal lineages within this group (Figure 5). In the cytochrome *b* network, only two Himalayan wolf haplotypes were recovered in the literature to date, one of which matched the Bhutan haplotype (Figure 6). Their intermediate position between wolf haplotypes and the ancestrally reconstructed nodes linking coyote and Tibetan fox may be consistent with a basal split within a broader canid phylogeny.

## 4 Discussion

Our mtDNA results suggest that wolves in Bhutan belong to the Himalayan wolf lineage. Across 13 amplified scat samples from two protected areas (JDNP and WCNP), D‐loop (*n* = 12) and cytochrome *b* (*n* = 11) sequences were identical and clustered within the monophyletic clade representing the Himalayan wolf. Haplotype‐network analysis provided concordant support for this placement. Together, these phylogenetic and network‐based inferences provide strong evidence that wolves in Bhutan's high‐altitude landscapes belong to the Himalayan wolf clade. However, further in‐depth analysis using nuclear markers is needed to more precisely assess their phylogeographic placement and population status.

Haplotype composition further supports this assignment and suggests mitochondrial lineage continuity of the Himalayan wolf within Bhutan and across the central Himalaya. Cytochrome *b* was invariant (*Himalayan wolf Cytochrome B 1*; KY996533) across all sequenced samples (*n* = 11), whereas the D‐loop revealed two haplotypes: *Himalayan wolf D‐loop 2* (NCBI GenBank Accession KY996530; *n* = 5, including the single sample from JDNP) and *HW9_Museum Nepal* (NCBI GenBank Accession AY333738; *n* = 7). Both D‐loop haplotypes were present within WCNP, and haplotype *D‐loop 2* was shared between the two parks. All haplotypes matched those previously reported from northwestern Nepal (Sharma et al. 2004; Werhahn et al. 2017), indicating that Bhutanese wolves fall within the known mtDNA diversity of Himalayan wolves and form a distinct clade. Although this haplotype sharing is consistent with shared maternal ancestry and plausibly reflects historical and/or current connectivity via movement along the Himalayan mountain range, inference is limited by the single JDNP sample, the maternal inheritance of mtDNA, and potential non‐independence of scat samples in the absence of individual identification. Nuclear markers will be required to evaluate contemporary gene flow and fine‐scale structure.

The absence of novel mtDNA haplotypes in this dataset should be interpreted cautiously and is not unexpected given the small sample size and the number of markers used. First, the dataset presented here is small and spatially unbalanced (with only one successfully amplified sample from JDNP), which limits the power to detect rare or localised haplotypes, particularly if mtDNA diversity is low or unevenly distributed. Second, scat‐based sampling can inadvertently oversample the same individuals, inflate the apparent frequency of common haplotypes and reduce the effective sample size when individual identity is unknown. Third, cytochrome *b* is relatively conserved and was not fully sequenced, and even in the D‐loop, short fragments typical of non‐invasive datasets have led to fewer observed substitutions, limiting the resolution needed to distinguish closely related maternal variants. Consequently, additional sampling across a broader set of localities, coupled with individual identification, full mitogenomes and higher‐resolution nuclear genomic data, will be necessary to more comprehensively characterise the genetic diversity of wolves in Bhutan. More comprehensive sampling will be required to test whether any Bhutan‐specific haplotypes occur at low frequency or in unsampled regions, an expectation consistent with broader Himalayan wolf mtDNA datasets generated using comparable short D‐loop and cytochrome *b* fragments (Werhahn et al. 2018).

Importantly, the limited mtDNA haplotype richness detected in Bhutan aligns with patterns reported across the Himalayan wolf range when comparable short mtDNA fragments are analysed. In Nepal, extensive non‐invasive sampling (scat and hair) using short D‐loop and cytochrome *b* fragments recovered low haplotype richness within regions: Humla had 3 haplotypes, Dolpa had 3 haplotypes and Kanchenjunga Conservation Area had 1 haplotype, despite large sample sizes (D‐loop sequences: Humla *n* = 72, Dolpa *n* = 89, KCA *n* = 13; cytochrome *b* subsets: Humla *n* = 24, Dolpa *n* = 16, KCA *n* = 7) (Werhahn et al. 2018). Similarly, in India, analysis of a reported 225‐bp D‐loop fragment from 19 wild‐collected samples identified only two haplotypes in the focal dataset (including one widespread haplotype shared with the Qinghai–Tibetan Plateau), and the expanded combined dataset (including published sequences) revealed a broader but still geographically structured pool of haplotypes with both shared and private variants (Joshi et al. 2020). Collectively, these studies indicate that Himalayan wolves often exhibit relatively low mtDNA haplotype richness in short fragments across large spatial scales, suggesting that modest sampling in Bhutan would be expected to recover previously documented haplotypes, even if additional rare or localised variants occur and remain undetected.

In Nepal, the Himalayan wolf population is expected to be very low, and the species is nationally categorised as Critically Endangered (Jnawali et al. 2011). In India, Shrotriya (2020) estimated 378–630 total individuals in the Indian Himalaya. For Bhutan, baseline information remains limited. Existing camera‐trap records indicate that wolves occur mainly in central Bhutan, with additional records in the JKSNR and the PFD to the west; however, these areas were not represented in our genetic sampling. In this context, our mtDNA evidence supports the interpretation that Bhutan's high‐altitude wolves are part of the Himalayan wolf lineage and that maternal lineages are not strongly isolated at the scale sampled. This interpretation is based on our data showing that the sampled wolf population falls within the Himalayan wolf clade, along with observed haplotype sharing among wolves from WCNP and JDNP and published haplotypes from Nepal.

However, apparent gaps in suitable habitat between WCNP and JDNP, and between JDNP and JKSNR, highlight the possibility of connectivity constraints that cannot be evaluated with the present dataset (Dhendup et al. 2024). At the same time, these gaps may reflect limitations of the habitat suitability model rather than true barriers to dispersal and should be investigated further on the ground. Moreover, the absence of strong biogeographic barriers across this landscape could facilitate movement and contribute to the genetic homogeneity observed in our current analysis. Expanded, systematically designed non‐invasive sampling across Bhutan's alpine and subalpine landscapes, prioritising JKSNR and PFD as well as potential intervening habitat gaps, is therefore needed to establish a national genetic baseline, identify potential connectivity bottlenecks and test whether these habitat discontinuities correspond to functional barriers. Integrating nuclear genotyping (to identify individuals and quantify gene flow) with targeted field surveys and human‐wolf conflict assessments will be essential to delineate conservation units, prioritise movement corridors among WCNP, JDNP and JKSNR, and guide evidence‐based national management of this evolutionarily distinct and threatened wolf lineage in Bhutan. We further recommend expanding sampling to include neighbouring wolf populations in Arunachal Pradesh on the Indian side, particularly the Tawang region, and on the adjacent Tibetan Plateau in the Tibet Autonomous Region of China. Including these populations will strengthen inferences about connectivity and lineage boundaries and further underscore the need for coordinated transboundary policies to ensure long‐term conservation of Himalayan wolves.

## Author Contributions

**Tashi Dhendup:** conceptualisation (equal), formal analysis (equal), funding acquisition (lead), investigation (equal), project administration (equal), writing – original draft (lead), writing – review and editing (equal). **Antonio Sampedro:** conceptualisation (equal), formal analysis (lead), investigation (equal), writing – original draft (supporting), writing – review and editing (equal). **Joshua J. Robinson:** conceptualisation (equal), data curation (lead), formal analysis (supporting), investigation (equal), writing – review and editing (equal). **Jan E. Janečka:** conceptualisation (equal), formal analysis (supporting), investigation (supporting), supervision (equal), writing – review and editing (equal). **Geraldine Werhahn:** conceptualisation (equal), investigation (equal), supervision (equal), writing – original draft (equal), writing – review and editing (equal).

## Ethics Statement

The authors have nothing to report.

## Conflicts of Interest

The authors declare no conflicts of interest.

## Supporting information


**Table S1:** GenBank reference sequences used in this study, with corresponding accession numbers.
**Table S2:** Samples collected in this study, with associated reference mitochondrial sequences for mitochondrial cytochrome b (MT‐*CYB*) and control region (MT‐CR), corresponding NCBI GenBank accession numbers and source citations.

## Data Availability

The sequences generated in this study matched previously published haplotypes; the corresponding GenBank accession numbers are provided in Table S2.
