# Supplementary material for: Wolves in Bhutan Are Part of the Ancient Himalayan Wolf (Canis lupus chanco) Lineage
Source: Ecol Evol. 2026 Aug 19;16(8):e74201. doi: 10.1002/ece3.74201 (PMC13488793; doi:10.1002/ece3.74201)
Supplement: Supplementary file 1 [file ECE3-16-e74201-s001.docx]

**Supplementary Materials**

**Wolves in Bhutan are part of the Himalayan wolf (*Canis lupus chanco*) lineage**

Tashi Dhendup, Antonio Sampedro, Joshua J Robinson, Jan E. Janecka, Geraldine Werhahn

**Table S1***.* GenBank reference sequences used in this study, with corresponding accession numbers.

| **Sample** | **Region** | **Species** | **Location** | **Accession** |
| --- | --- | --- | --- | --- |
| JACKAL1 | MT-*CR* | *Canis aureus* | Turkey | [KT988007.1](https://www.ncbi.nlm.nih.gov/nuccore/KT988007.1) |
| JACKAL2 | MT-*CR* | *Canis aureus* | Turkey | [KT988009.1](https://www.ncbi.nlm.nih.gov/nuccore/KT988009.1) |
| HW9_Museum_Nepal | MT-*CR* | *Canis lupus chanco* | Museum (Nepal) | [AY333738](https://www.ncbi.nlm.nih.gov/nuccore/AY333738) |
| HW7_Museum_Tibet | MT-*CR* | *Canis lupus chanco* | Museum (Tibet) | [AY333739](https://www.ncbi.nlm.nih.gov/nuccore/AY333739) |
| HW4_Ladhak | MT-*CR* | *Canis lupus chanco* | Ladhak | [AY333741](https://www.ncbi.nlm.nih.gov/nuccore/AY333741) |
| HW10_Museum_Tibet | MT-*CR* | *Canis lupus chanco* | Museum (Tibet) | [AY333742](https://www.ncbi.nlm.nih.gov/nuccore/AY333742) |
| HW6_Qinghai | MT-*CR* | *Canis lupus chanco* | Qinhai_lake | [JX415345](https://www.ncbi.nlm.nih.gov/nuccore/JX415345) |
| HW5_Qinghai | MT-*CR* | *Canis lupus chanco* | Qinhai_lake | [JX415347](https://www.ncbi.nlm.nih.gov/nuccore/JX415347) |
| HW11_Qinghai | MT-*CR* | *Canis lupus chanco* | Qinhai_lake | [JX415348](https://www.ncbi.nlm.nih.gov/nuccore/JX415348) |
| HW8_Tibet | MT-*CR* | *Canis lupus chanco* | Tibet | [KF573616](https://www.ncbi.nlm.nih.gov/nuccore/KF573616) |
| HW1_Humla_NP | MT-*CR* | *Canis lupus chanco* | Humla | [KY996529](https://www.ncbi.nlm.nih.gov/nuccore/KY996529) |
| HW2_Humla_NP | MT-*CR* | *Canis lupus chanco* | Humla | [KY996530](https://www.ncbi.nlm.nih.gov/nuccore/KY996530) |
| Dog_Nepal | MT-*CR* | *Canis lupus familiaris* | Nepal | [KY996526](https://www.ncbi.nlm.nih.gov/nuccore/KY996526) |
| GW_Spain | MT-*CR* | *Canis lupus lupus* | Spain | [DQ480505](https://www.ncbi.nlm.nih.gov/nuccore/DQ480505) |
| GW_Saudi_Arabia | MT-*CR* | *Canis lupus lupus* | Saudi Arabia | [DQ480506](https://www.ncbi.nlm.nih.gov/nuccore/DQ480506) |
| GW_Sweeden | MT-*CR* | *Canis lupus lupus* | Sweeden | [KF661040](https://www.ncbi.nlm.nih.gov/nuccore/KF661040) |
| GW_China_1 | MT-*CR* | *Canis lupus lupus* | China | [KF661041](https://www.ncbi.nlm.nih.gov/nuccore/KF661041) |
| GW_Israel | MT-*CR* | *Canis lupus lupus* | Israel | [KF661042](https://www.ncbi.nlm.nih.gov/nuccore/KF661042) |
| GW_Poland | MT-*CR* | *Canis lupus lupus* | Poland | [KF661045](https://www.ncbi.nlm.nih.gov/nuccore/KF661045) |
| GW_Russia | MT-*CR* | *Canis lupus lupus* | Russia | [KF661046](https://www.ncbi.nlm.nih.gov/nuccore/KF661046) |
| GW_Italy | MT-*CR* | *Canis lupus lupus* | Italy | [KF661048](https://www.ncbi.nlm.nih.gov/nuccore/KF661048) |
| GW_Iran | MT-*CR* | *Canis lupus lupus* | Iran | [KF661051](https://www.ncbi.nlm.nih.gov/nuccore/KF661051) |
| GW_Alaska_1 | MT-*CR* | *Canis lupus lupus* | Alaska | [KF661058](https://www.ncbi.nlm.nih.gov/nuccore/KF661058) |
| GW_Canada_2 | MT-*CR* | *Canis lupus lupus* | Canada | [KF661061](https://www.ncbi.nlm.nih.gov/nuccore/KF661061) |
| GW_Alaska_2 | MT-*CR* | *Canis lupus lupus* | Alaska | [KF661066](https://www.ncbi.nlm.nih.gov/nuccore/KF661066) |
| GW_Canada_1 | MT-*CR* | *Canis lupus lupus* | Canada | [KF661074](https://www.ncbi.nlm.nih.gov/nuccore/KF661074) |
| GW_Mongolia_1 | MT-*CR* | *Canis lupus lupus* | Mongolia | [KY996527](https://www.ncbi.nlm.nih.gov/nuccore/KY996527) |
| GW_Mongolia_2 | MT-*CR* | *Canis lupus lupus* | Mongolia | [KY996528](https://www.ncbi.nlm.nih.gov/nuccore/KY996528) |
| IW_2 | MT-*CR* | *Canis lupus pallipes* | India | [AY289973](https://www.ncbi.nlm.nih.gov/nuccore/AY289973) |
| IW_1 | MT-*CR* | *Canis lupus pallipes* | India | [AY333745](https://www.ncbi.nlm.nih.gov/nuccore/AY333745) |
| HW_CytB1 | MT-*CYB* | *Canis lupus chanco* | Humla | [KY996533](https://www.ncbi.nlm.nih.gov/nuccore/KY996533) |
| HW_CytB2 | MT-*CYB* | *Canis lupus chanco* | Humla | [KY996534](https://www.ncbi.nlm.nih.gov/nuccore/KY996534) |
| Dog_Nepal | MT-*CYB* | *Canis lupus familiaris* | Nepal | [KY996532](https://www.ncbi.nlm.nih.gov/nuccore/KY996532) |
| IW_1 | MT-*CYB* | *Canis lupus pallipes* | India | [AY291432](https://www.ncbi.nlm.nih.gov/nuccore/AY291432) |
| GW_Israel_1 | MT-*CYB* | *Canis lupus lupus* | Israel | [KT447705](https://www.ncbi.nlm.nih.gov/nuccore/KT447705) |
| GW_Canada_1 | MT-*CYB* | *Canis lupus lupus* | Canada | [KT447699](https://www.ncbi.nlm.nih.gov/nuccore/KT447699) |
| GW_Saudi_Arabia_1 | MT-*CYB* | *Canis lupus lupus* | S. Arabia | [KT447704](https://www.ncbi.nlm.nih.gov/nuccore/KT447704) |
| GW_Saudi_Arabia_2 | MT-*CYB* | *Canis lupus lupus* | S. Arabia | [DQ480506](https://www.ncbi.nlm.nih.gov/nuccore/DQ480506) |
| GW_Russia | MT-*CYB* | *Canis lupus lupus* | Russia | [DQ480503](https://www.ncbi.nlm.nih.gov/nuccore/DQ480503) |
| Tibetan_Fox | MT-*CYB* | *Vulpes ferrilata* |  | [KT033906](https://www.ncbi.nlm.nih.gov/nuccore/KT033906) |
| Coyote1 | MT-*CYB* | *Canis latrans* | USA | [KT447695](https://www.ncbi.nlm.nih.gov/nuccore/KT447695) |

**Table S2.** Samples collected in this study, with associated reference mitochondrial sequences for mitochondrial cytochrome b (MT-*CYB*) and control region (MT-CR), corresponding NCBI GenBank accession numbers, and source citations.

| **Sample** | **Species** | **Reference Cyt-B** | **Reference NCBI GenBank Accession** | **Reference** | **Reference D-loop** | **Reference NCBI GenBank Accession** | **Reference** |
| --- | --- | --- | --- | --- | --- | --- | --- |
| JDNP103 | *Canis lupus chanco* | Himalayan wolf Cytochrome B 1 | [KY996533](https://www.ncbi.nlm.nih.gov/nuccore/KY996533) | Werhahn et al. (2017) | Himalayan wolf D-loop 2 | [KY996530](https://www.ncbi.nlm.nih.gov/nuccore/KY996530) | Werhahn et al. (2017) |
| WCNP014 | *Canis lupus chanco* | Himalayan wolf Cytochrome B 1 | [KY996533](https://www.ncbi.nlm.nih.gov/nuccore/KY996533) | Werhahn et al. (2017) | NA |  |  |
| WCNP016 | *Canis lupus chanco* | Himalayan wolf Cytochrome B 1 | [KY996533](https://www.ncbi.nlm.nih.gov/nuccore/KY996533) | Werhahn et al. (2017) | HW9_Museum Nepal | [AY333738](https://www.ncbi.nlm.nih.gov/nuccore/AY333738) | Sharma et al. (2004) |
| WCNP017 | *Canis lupus chanco* | NA |  |  | HW9_Museum Nepal | [AY333738](https://www.ncbi.nlm.nih.gov/nuccore/AY333738) | Sharma et al. (2004) |
| WCNP028 | *Canis lupus chanco* | Himalayan wolf Cytochrome B 1 | [KY996533](https://www.ncbi.nlm.nih.gov/nuccore/KY996533) | Werhahn et al. (2017) | Himalayan wolf D-loop 2 | [KY996530](https://www.ncbi.nlm.nih.gov/nuccore/KY996530) | Werhahn et al. (2017) |
| WCNP033 | *Canis lupus chanco* | NA |  |  | Himalayan wolf D-loop 2 | [KY996530](https://www.ncbi.nlm.nih.gov/nuccore/KY996530) | Werhahn et al. (2017) |
| WCNP036 | *Canis lupus chanco* | Himalayan wolf Cytochrome B 1 | [KY996533](https://www.ncbi.nlm.nih.gov/nuccore/KY996533) | Werhahn et al. (2017) | Himalayan wolf D-loop 2 | [KY996530](https://www.ncbi.nlm.nih.gov/nuccore/KY996530) | Werhahn et al. (2017) |
| WCNP041 | *Canis lupus chanco* | Himalayan wolf Cytochrome B 1 | [KY996533](https://www.ncbi.nlm.nih.gov/nuccore/KY996533) | Werhahn et al. (2017) | HW9_Museum Nepal | [AY333738](https://www.ncbi.nlm.nih.gov/nuccore/AY333738) | Sharma et al. (2004) |
| WCNP047 | *Canis lupus chanco* | Himalayan wolf Cytochrome B 1 | [KY996533](https://www.ncbi.nlm.nih.gov/nuccore/KY996533) | Werhahn et al. (2017) | HW9_Museum Nepal | [AY333738](https://www.ncbi.nlm.nih.gov/nuccore/AY333738) | Sharma et al. (2004) |
| WCNP057 | *Canis lupus chanco* | Himalayan wolf Cytochrome B 1 | [KY996533](https://www.ncbi.nlm.nih.gov/nuccore/KY996533) | Werhahn et al. (2017) | Himalayan wolf D-loop 2 | [KY996530](https://www.ncbi.nlm.nih.gov/nuccore/KY996530) | Werhahn et al. (2017) |
| WCNP069 | *Canis lupus chanco* | Himalayan wolf Cytochrome B 1 | [KY996533](https://www.ncbi.nlm.nih.gov/nuccore/KY996533) | Werhahn et al. (2017) | HW9_Museum Nepal | [AY333738](https://www.ncbi.nlm.nih.gov/nuccore/AY333738) | Sharma et al. (2004) |
| WCNP070 | *Canis lupus chanco* | Himalayan wolf Cytochrome B 1 | [KY996533](https://www.ncbi.nlm.nih.gov/nuccore/KY996533) | Werhahn et al. (2017) | HW9_Museum Nepal | [AY333738](https://www.ncbi.nlm.nih.gov/nuccore/AY333738) | Sharma et al. (2004) |
| WCNP089 | *Canis lupus chanco* | Himalayan wolf Cytochrome B 1 | [KY996533](https://www.ncbi.nlm.nih.gov/nuccore/KY996533) | Werhahn et al. (2017) | HW9_Museum Nepal | [AY333738](https://www.ncbi.nlm.nih.gov/nuccore/AY333738) | Sharma et al. (2004) |
